# *QuickStats:* Number of Homicides Committed, by the Three Most Common Methods* — United States, 2010–2016

**DOI:** 10.15585/mmwr.mm6729a4

**Published:** 2018-07-27

**Figure:**
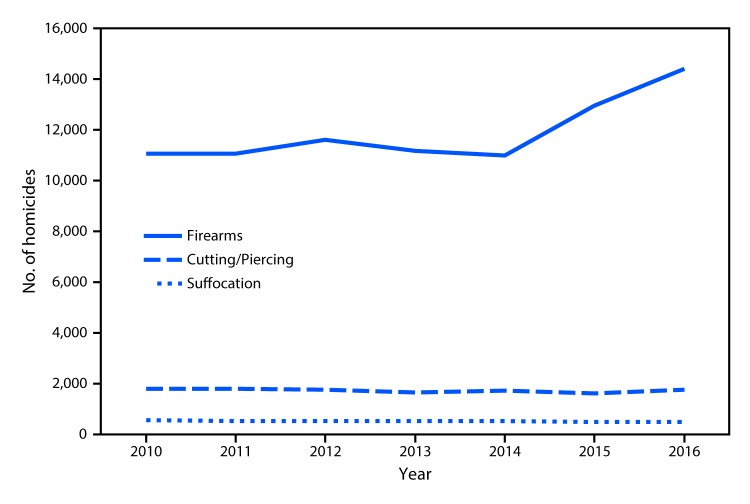
During 2010–2016, use of firearms was the most common method in the United States, followed by the use of instruments for cutting and piercing and then suffocation. The number of firearm-related homicides was relatively stable during 2010–2014 (fluctuating between 11,008 and 11,622) but then increased by 31% from 2014 (11,008) to 2016 (14,415). In 2016, the number of homicides involving firearms was approximately eight times the number of those involving cutting and piercing (1,781) and approximately 30 times those involving suffocation (502).

**Source:** National Vital Statistics System, Underlying Cause of Death Data, 2000–2016. https://wonder.cdc.gov/ucd-icd10.html.

